# Annotations

**Published:** 1887-02-26

**Authors:** 


### Annotations.

Provident Dispensaries.

Few, perhaps, of our modern charitable institutions are more praiseworthy in their methods of operation, and more likely to raise the moral tone of their beneficiaries by encouraging independence and thrift, than the Provident Dispensaries. Many, if not all, of these co-operative societies (for such they really are, though perhaps in a somewhat modified degree) are formed with the intention of enabling the earners of low or insufficient wages to obtain good medical attendance, and such drugs as may be necessary, whenever they are out of health, by joining such an association, and paying a small weekly or monthly sum in advance. Thus, at one provident dispensary with which we are acquainted, if a man or woman pays sixpence, or a man and wife pay tenpence per month, regularly in advance, they become entitled to attendance and treatment at the hands of the dispensary medical officer, who must be legally qualified for his post, and also to medicine, without further charge, whenever ill-health may overtake or accident befall them. That terror of incurring a long doctor's bill, which amongst poor families so often leads to the long neglect of the earlier symptoms of illness, and the fighting against its attacks, until perhaps a chronic or fatal sickness is produced, which might have been cured in its first stages, has no reason for its existence amongst the members of a provident dispensary. Such an institution is in fact a mutual insurance society, which provides the workers of the community with ample and comforting certainty, that whatever form of sickness may happen to fall upon them can be met and combated from the beginning, without bringing in its train the terrible incubus of a heavy doctor's bill, and the consequent tax upon wages as yet unearned.

Aurard's Incubator.

In these days of invention and improvements upon nature, the use of artificial means of hatching, or ensuring the vitality of newly hatched chickens, is well known to the public, and almost every poultry-lover has his or her pet form of incubator. The introduction of a human incubator, one of the latest developments of medical science, will, however, we venture to think, prove a novel idea to many of our readers. This instrument was invented by Dr. Aurard, of Paris, and is intended for the rearing of children prematurely born, who, if subjected to ordinary methods only, would probably not survive their birth more than an hour or two. It consists of a wooden box, divided into two compartments, an upper and a lower, with free communication between them. The child is placed in the upper compartment, the lower being occupied by hot bottles, which radiate heat to a carefully-regulated extent, and so raise the temperature to the necessary degree of warmth. Fresh air is only communicated to the child through this lower compartment, whilst the exhausted air is allowed to escape through a specially contrived cutlet in the upper part of the structure. To furnish the necessary moisture to the atmosphere a wet sponge is also placed in the lower compartment. Such an incubator is, we believe, now in use at the Glasgow Maternity Hospital, for the rearing of triplet children who were recently born there, but we do not yet know how far the experiment is likely to prove a success in this particular instance.

Town *v.* Country.

"Enquirer" wants to know if it be true, as is generally supposed, that aged people in the country are more robust, and live longer than in towns? It is quite true, and very much more that bears on the same question of town or country is also true. Everybody flocks into towns nowadays. What can be the reason? Town people are neither so healthy nor so happy as country people. The struggle for existence is severer in towns. Everything is narrower, more limited, more confined. There is no sense of space, of freedom, of infinity. Those who interest themselves in the matter may be assured that the growth of town populations at the expense of the rural districts means the physical deterioration of the whole population. But the evil does not stop there. Mental and moral deterioration proceed *pari passu* with physical. Town people, and especially Londoners, pride themselves upon their mental superiority to the rustic. There is not a shadow of foundation for any such idea. In superficial smartness the town man may make the better show; but in practical sense and all-round capacity he is a mere child compared with the countryman. Transport the two into a new country, under entirely new circumstances, and see which of them will the more readily and practically adapt himself to his surroundings. Whilst the town man is bemoaning his fate, and whimpering over the loss of his music-hall and his morning rasher, the countryman is tucking up his sleeves, and digging out a patch for potatoes, or roofing-in his log-hut. England is one of the finest countries in the world for agricultural occupation, and ought to possess a numerous, prosperous, and contented peasantry. It says little for the statesmanship of both political parties, that the peasant's life is fast becoming insupportable and impossible.

---

It is curious to observe how custom has crystallised and made general the use of particular adjectives in the qualification of the sum of our benevolence to the hospitals. A gift of ten pounds is a "liberal donation," a hundred pounds is a "handsome donation," a thousand pounds is a "munificent donation," while ten thousand pounds is a "princely donation" The Guys and the Hunts are so rarely found, tha no adjective has been left unappropriated for the designation of donations of a larger sum than the last named.

